# Evaluation of dose-response relationships between smoking tobacco, alcohol consumption and oral cancer: a systematic review and meta-analysis

**DOI:** 10.1186/s12889-026-27796-1

**Published:** 2026-05-22

**Authors:** G. Aswathi, Andre Carvalho, Sathishrajaa Palaniraja, M. Shivacharan, Bhukya Nom Kumar Naik, Saman Warnakulasuriya, Kishore Chaudhry, Muralidhar Kulkarni, Ranjitha S. Shetty, Shirley Lewis, Vijay Shree Dhyani, Suzanne Tanya Nethan, Ravivarman Lakshmanasamy, Vasudeva Guddattu, K. Devaraja

**Affiliations:** 1 Department of Head and Neck Surgery, Kasturba Medical College, Manipal Academy of Higher Education, Manipal, India; 2 Early Detection, Prevention, and Infections Branch (EPR), International Agency for Research on Cancer [IARC/WHO], Lyon, France; 3 International Agency for Research on Cancer [IARC/WHO], Lyon, France; 4 Department of Radiation Oncology, Kasturba Medical College, Manipal Academy of Higher Education, Manipal, India; 5 Department of Anatomy, Kasturba Medical College, Manipal Academy of Higher Education, Manipal, India; 6 Faculty of Health/School of Nursing & Midwifery/Institute for Health Transformation, Deakin University, Geelong, VIC Australia; 7 Department of Oral Medicine & Experimental Pathology, King’s College London, London, UK; 8 Las Vegas, Nevada USA; 9 Department of Community Medicine, Kasturba Medical College, Manipal Academy of Higher Education, Manipal, India; 10 Kasturba Medical College, Manipal Academy of Higher Education, Manipal, India; 11 Department of Applied Statistics and Data Science, Prasanna School of Public Health, Manipal Academy of Higher Education, Manipal, India

**Keywords:** Oral cancer, Dose-response, Tobacco smoking, Alcohol consumption, Meta-analysis, Risk factors

## Abstract

**Background:**

Tobacco smoking and alcohol consumption are established independent risk factors for oral carcinogenesis, yet the quantitative dose-response relationships for key exposure variables remain unclear.

**Methods:**

A systematic review and dose-response meta-analysis (PROSPERO registration number: CRD42024531040) was conducted following the PRISMA 2020 guidelines. Cohort, case-control, and cross-sectional studies reporting relative risks (RRs) or odds ratios for at least one of the exposure variables, such as smoking frequency, smoking duration, alcohol duration, and age at smoking initiation were included. Random-effects dose-response models with restricted cubic splines were applied. Quality of the included studies was assessed using the Newcastle-Ottawa Scale.

**Results:**

Data from 34 eligible studies (27 on smoking frequency, 22 on smoking duration, 11 on alcohol duration, and 11 on age at smoking initiation) were analysed. Smoking frequency showed a strong non-linear association, peaking at 30 smoking units/day (RR: 5.98; 95% CI: 3.37–10.60). Smoking duration was positively associated with risk, reaching a maximum at 40 years (RR: 2.19; 95% CI: 1.48–3.23). Duration of alcohol consumption showed a positive but weaker association, with the highest risk occurring at 50 years (RR: 3.13; 95% CI: 1.40–6.97). Age at smoking initiation was not independently associated with risk.

**Conclusions:**

Smoking frequency and duration each exhibit strong and independent dose-response relationships with oral cancer, while alcohol duration independently confers moderate risk. These findings highlight exposure-specific risk thresholds that can inform targeted prevention and screening strategies, particularly in populations with high incidence rates.

**Supplementary Information:**

The online version contains supplementary material available at 10.1186/s12889-026-27796-1.

## Introduction

The global burden of oral cancer is substantial, with the age-standardised incidence rate of 4 per 100,000 [1]. Worldwide, oral cancer represents a significant portion of cancer incidence, with rising trends observed in regions such as Southeast Asia and parts of Europe [2, 3]. This alarmingly high prevalence is largely attributable to alcohol and tobacco use, particularly in the form of smoking tobacco, smokeless tobacco (SLT), betel nut/quid chewing with or without tobacco, and alcohol [4, 5].

Tobacco and alcohol consumption have long been recognised as major modifiable risk factors for oral cancer [6]. Tobacco, whether smoked or chewed, contains numerous carcinogenic compounds that lead to genetic aberrations, resulting in oral cancer [7]. Cigarettes contain more than 7,000 chemicals, many of which are toxic and at least 70 are established carcinogens [8, 9]. Tobacco smoke also comprises a complex mixture of reactive oxygen and nitrogen species that can damage key macromolecules, including lipids, proteins, and nucleic acids [8]. Accumulating evidence highlights the role of smoking-induced oxidative stress in promoting chronic inflammation and carcinogenesis, providing mechanistic support for the observed epidemiological associations [8]. Compared with non-smokers, people who smoke face a substantially higher risk of oral cancer mortality, and the risk rises progressively with the number of cigarettes smoked per day [9]. Overall, smokers have approximately a five- to tenfold greater risk of developing oral cancer [9]. This risk is further amplified when smoking is combined with alcohol consumption [10–12]. Biological mechanisms support this association, as alcohol-dependent individuals with oropharyngeal cancer have been shown to exhibit markedly elevated salivary acetaldehyde concentrations, potentially due to concurrent smoking and poor oral hygiene [13]. Smoking can rapidly alter the oral microbial environment, shifting the flora toward Gram-positive bacteria and increasing acetaldehyde production by approximately 50–60% compared with non-smokers [14]. Globally, alcohol consumption was estimated to account for approximately 75,000 cases of oral cavity cancer in 2020, underscoring its substantial contribution to disease burden [15].

Although existing studies have consistently identified and established tobacco and alcohol as risk factors for oral cancer, the variations in population demographics and level of exposure to these carcinogens create challenges in forming a consensus regarding the magnitude of risk posed by each exposure variable. A quantified dose-response analysis is crucial to understand the risk of oral cancer at various exposure levels to the carcinogen. This information is pivotal for accurately estimating the oral cancer burden at the individual level, through statistical models assessing the risk and at the population level to shape screening strategies, especially in populations with high exposure rates [16]. The objective of this meta-analysis is to provide a comprehensive quantification of the association between various smoking and alcohol related exposure variables and the risk of developing oral cancer through a dose-response meta-analysis. We aim to present risk curves for each variable independently, illustrating the dose-response relationships in terms of daily frequency and duration (in years) of consumption along with the age at initiation of carcinogen exposure, thereby offering critical insights into their impact on the risk of developing oral cancer.

## Methodology

This systematic review and dose-response meta-analysis was conducted following the Preferred Reporting Items for Systematic reviews and Meta-Analyses (PRISMA) 2020 guidelines [17] and was prospectively registered in International Prospective Register of Systematic Reviews (PROSPERO): CRD42024531040.

### Search strategy

A comprehensive search of PubMed, EMBASE, Scopus, Web of Science, Cochrane Central, and ProQuest was performed from inception to March 2025. The search combined controlled vocabulary and free-text terms related to oral cancer, tobacco smoking, alcohol consumption, and risk estimates. The search strategy for one of the databases is available as the supplementary material. The reference lists of the included articles and related reviews were manually checked to identify any further studies that met the eligibility criteria.

### Eligibility criteria

We included observational studies (case-control, cohort, and cross-sectional designs) published in English that reported relative risks (RRs) or odds ratios (ORs) with 95% confidence intervals (CIs) for at least one of the following quantitative exposure variables:

- Smoking frequency: number of smoked tobacco product/s per day (units/day)
- Smoking duration (years)
- Alcohol consumption duration (years)
- Age at smoking initiation (Age in years)

Dose-response models were constructed using study-specific risk estimates comparing quantitative exposure levels among current (or combined current/ever) users with never users as the reference category. Studies focusing on passive smoking or former users were excluded. Given the rarity of oral cancer, odds ratio and relative risk (RR) were considered equivalent measures of risk [18].

### Study selection and data extraction

Two reviewing teams (team 1 included authors, GA and VSD and team 2 included BNKN and MS) independently screened titles, abstracts, and full texts for eligibility using the Rayyan Software. Discrepancies were resolved through discussion with a third reviewer (KD). For each included study, the following data were extracted: author, year, country, study design, sample size, number of cases/controls, exposure variable definitions, type of tobacco or alcohol product, adjustment variables, and reported effect estimates. When both adjusted and unadjusted estimates were available, the adjusted values were used. If only raw data were provided, unadjusted RRs were calculated.

### Exposure quantification

Effect estimates and 95% CIs were log-transformed to obtain log (RR) values and their variances. For closed exposure intervals, midpoints were calculated as the average of the lower and upper bounds. For open-ended categories, the midpoint was estimated by multiplying the lower bound by 1.2 (upper-open intervals) or dividing the upper bound by two (lower-open intervals) [19–21].

### Quality assessment

The Newcastle-Ottawa Scale (NOS) [22, 23] was used to assess methodological quality, evaluating selection, comparability, and exposure/outcome domains. Two reviewing teams scored each study independently, with disagreements resolved by consensus.

### Statistical analysis

A dose-response meta-analysis was conducted using generalised least squares regression to model both linear and non-linear associations between exposure and oral cancer risk [24]. Restricted cubic splines with three knots were applied to flexibly model potential non-linear trends. Separate models were run for each exposure variable. The model with the lowest Akaike information criterion (AIC) value was chosen as the best fit. Departure from linearity was assessed by comparing the restricted cubic spline model with the linear model using likelihood-based criteria and Wald-type tests.

Between-study heterogeneity was quantified using the residual Q-statistic (QE) with corresponding p-values and the I² statistic, representing the proportion of total variability attributable to between-study differences [21]. Publication bias was assessed via funnel plot inspection and Kendall’s tau rank correlation. Sensitivity analyses were performed using a leave-one-out approach to assess the influence of individual studies.

Statistical analyses were carried out using R software (v4.3.1), employing the dosresmeta, meta, and metafor packages to perform the required computations [24].

## Results

### Study selection

**Fig. 1:**
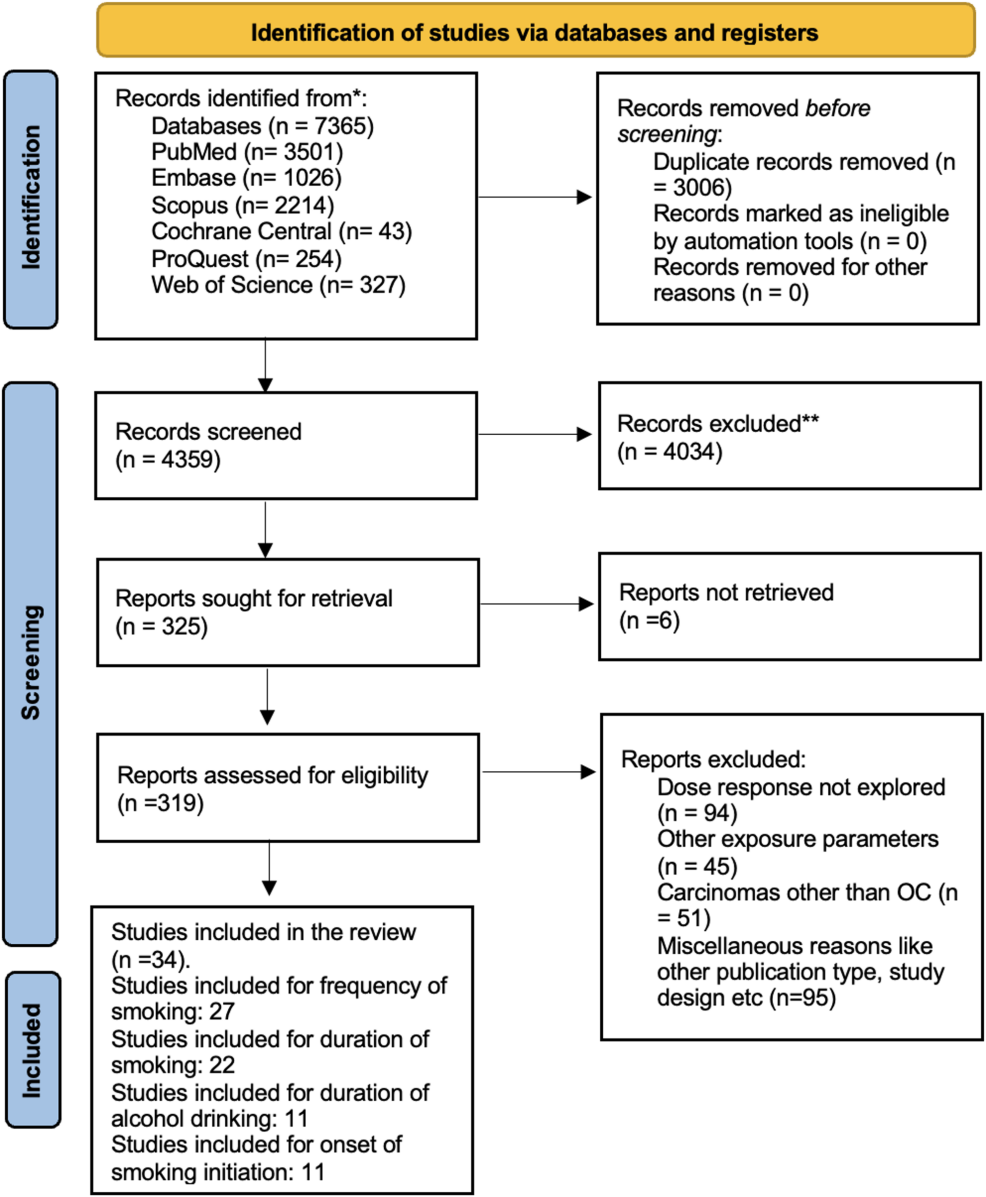
PRISMA 2020 flow diagram

The search yielded 7,365 records, of which 3,006 duplicates were removed. After screening 4,359 titles and abstracts, 319 full-text articles were assessed for eligibility. Thirty-four studies met the inclusion criteria (31 case-control studies, 2 cohort studies and 1 cross-sectional study) (Fig. 1). The study characteristics are summarised in Table 1.

**Table 1:** Study characteristics

**Matched Case–Control Studies**									
**Study**	**Region**	**Gender**	**Cases**	**Controls**	**Matching factors**	**Controlled variables**	**Exposure**	**Product**	**NOQAS**
Winn et al., 1981 [25]	USA	F	232	410	Age, race, ascertainment source, residence	Age, residence, race, ascertainment source	SF	Cigarette	5
Mashberg et al., 1981 [26]	USA	M	181	497	Age	Drinking habits, age	SF	Cigar, pipe	6
Sankarnarayanan et al., 1989 [2] [27]	India	M	158	314	Age, religion	Residual age effect, pan-tobacco chewing and alcohol drinking.	SF + SI + SD + AD	Bidi, cigarette; toddy, arrack	6
Franceschi et al., 1990 [28]	Italy	M	157	1272	Age, residence	Age, education, occupation, alcohol/smoking habits, residence.	SF + SI + SD + AD	Cigarette; wine, beer, liquor	6
Nandakumar et al., 1990 [29]	India	M&F	348	348	Age, sex, residence	Pan chewing	SF + SD	Cigarette, bidi	6
Mashberg et al., 1993 [30]	USA	M	392	2388	Age	Age, race, alcohol	SF + SD	Cigarette, cigar, pipe	7
Day et al., 1993 [31]	USA	M&F	871	979	Age, sex, race	Alcohol, age, sex, location, respondent status (subject versus proxy)	SF + SI + SD	Cigarette	7
Kabat et al., 1994 [32]	USA	M&F	1560	2930	Age, sex, race	Age, education, alcohol, race, time period, type of hospital.	SF + SD	Cigarette	6
Bundgard et al., 1995 [33]	Denmark	M&F	161	400	Age, sex	Lifetime tobacco and alcohol	SF	Cigarette	6
Zheng et al., 1997 [34]	China	M&F	111	111	Age, sex	Alcohol/tobacco smoking, education	SF + SI + SD + AD	Cigarette, cigar, pipe	6
De Stefani et al., 1998 [35]	Uruguay	M	206	427	Age, Residence, urban/rural	Age, residence, urban/rural status, birthplace, education, and total alcohol consumption.	SF + SD	Cigarette	6
Hayes et al., 1999 [36]	Puerto Rico	M&F	519	629	Age, sex	Age, alcohol use	SF	Cigarette	7
Balaram et al., 2002 [37]	India	M	591	582	Age, center	Education, tobacco chewing habit, alcohol, age, center.	SF + SI	Cigarette, bidi	6
Castellsague et al., 2004 [38]	Italy, Spain, Ireland, Poland, Cuba, Canada, India, Sudan and Australia	M&F	375	375	Age, sex	Center, gender, age group, years of schooling and average daily consumption of pure ethanol/ smoked per day	SF + SD + AD	Cigarette; alcohol	7
Llewellyn et al., 2004 [39]	England	M&F	116	207	Age, sex, residence	Alcohol	SF + SI + SD	Cigarette, cigar	6
Polosel et al., 2008 [40]	Italy and the Swiss Cantonof Vaud	M	314	1161	Age, residence, interview year	NM	SF	Cigarette	7
Muwonge et al., 2008 [12]	India	M&F	282	1410	Age, sex, panchayat, response status	Education, religion, alcohol/smoking, tobacco chewing habits.	SF + SD + AD	Cigarette, bidi	7
Fu et al., 2013 [41]	China	M	319	428	Age	Age, education, region	SF + SD	Cigarette	7
Amtha et al., 2014 [42]	Indonesia	M&F	81	162	Age, sex	Alcohol, betel quid chewing and dietary pattern.	SF + SD	Cigarette	6
C. J et al., 2018 [43]	India	M&F	200	200	Age, sex	NM	AD	Alcohol	5
Edirisinghe et al., 2023 [44]	Sri Lanka	M&F	105	210	Age, sex	NM	AD	Alcohol	6
Gholap et al., 2023 [45]	India	M	824	1206	Age, region	Age, residence, education, ethanol gram years, body mass index, hypertension, lifetime vegetarian status and second-hand smoke exposure.	SI	Cigarette, bidi	6

**Unmatched Case–Control Studies**									
**Study**	**Region**	**Gender**	**Cases**	**Controls**		**Controlled variables**	**Exposure**	**Product**	**NOQAS**
Sankarnarayanan et al., 1989 [1][46]	India	M	187	895		Age, smoking/ alcohol	SF + SI + SD + AD	Bidi, cigarette	6
Sankarnarayanan et al., 1990 [47]	India	M	414	895		Age, religion, pan chewing, alcohol, snuff.	SF + SD + AD	Bidi, cigarette	6
Idris et al., 1995 [48]	Sudan	M&F	Hospital: 375; Population:271	Hospital:204; Population:2820		Age, sex, tribe, residence	SD	Cigarette	6
Morenzo-Lopez et al., 2000 [49]	Spain	M&F	75	150		NM	SF	Cigarette, cigar	4
Dikshit et al., 2000 [50]	India	M	148	260		Age, tobacco quid chewing	SD	Cigarette, bidi	7
Znaor et al., 2003 [51]	India	M	1563	3638		Age, center, education, alcohol/smoking, chewing habit.	SF + SD + AD	Multiple	6
Guneri et al., 2005 [52]	Turkey	M&F	79	61		NM	SI	NM	4
De Stefani et al., 2007 [53]	Uruguay	M	335	1501		Age, residence, urban/rural status, hospital, year at diagnosis, education, family history of cancer among first-degree relatives,occupation, total vegetables and fruits consumption, mate´ intake, and alcohol drinking, smoking status, years since quit and cigarettes/day among current smokers.	SF + SD + AD	Cigarette	7
Siddiqui et al., 2024 [54]	Pakistan	M&F	111	132		NM	SF + SD	Cigarette	4

**Cohort Studies**									
**Study**	**Region**	**Gender**	**Sample size**			**Controlled variables**	**Exposure**	**Product**	**NOQAS**
Jayalekshmi et al., 2011 [55]	India	M	66277			Age, time, income, education	SF + SI + SD	Bidi	9
Maasland et al., 2014 [56]	Netherlands	M&F	110			Age, sex, alcohol	SF + SD	Cigarette	9

**Cross-sectional Study**									
**Study**	**Region**	**Gender**	**Cases**	**Controls**		**Controlled variables**	**Exposure**	**Product**	**NOQAS**
Evstifeeva et al., 1992 [57]	Uzbekistan	M	191	466		Nass, alcohol, age	SI	Cigarette	7

Based on the Newcastle-Ottawa Scale (NOS), studies scoring 0-3 stars were considered at high risk of bias, 4-6 stars at moderate risk of bias, and 7-9 stars at low risk of bias

*SF *Smoking frequency, *SD *Smoking duration, *SI *Smoking initiation age, *AD *Alcohol duration, *NOQAS *New-castle Ottawa Quality Assessment Scale, *M *Male, *F *Female, *OC *Oral cancer, *NM *Not Mentioned

### Smoking frequency and oral cancer risk

**Fig. 2:**
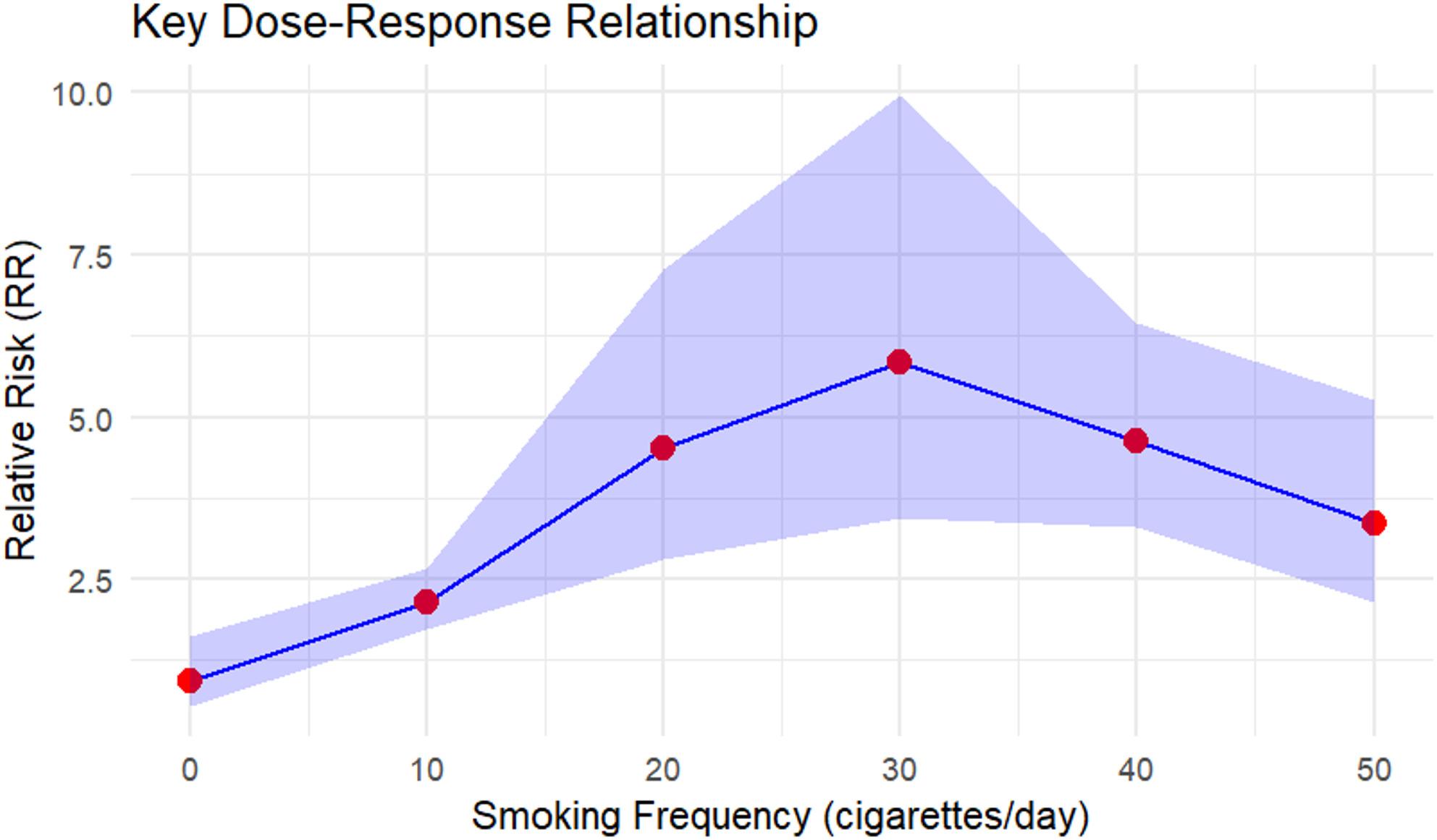
Dose-response relationship between smoking frequency and oral cancer risk. Shaded areas indicate 95% confidence intervals

Twenty-seven studies contributed to the dose-response analysis of smoking frequency. The restricted cubic spline model demonstrated improved fit in comparison with the linear model (AIC 205.6 vs. 212.6). Likelihood comparison indicated significant departure from linearity (χ² ≈ 8.9, *p* ≈ 0.003). The overall spline terms were statistically significant (QM = 15.99, df = 2, *p* = 0.0003). The predicted RR increased with frequency of smoking tobacco, peaking at 30 units/day (RR = 5.98; 95% CI: 3.37–10.60), followed by a gradual decline at higher doses (40 units/day: RR = 4.98; 95% CI: 3.49–7.13; 50 units/day: RR = 3.84; 95% CI: 2.41–6.13) (Fig. 2; Table 2). Substantial between-study heterogeneity was observed (QE [df = 85] = 468.58, *p* < 0.0001; I² = 81.9%). No evidence of publication bias was detected (Kendall’s tau = 0.087, *p* = 0.236).

**Table 2:** Predicted RR for the frequency of smoking and the risk of oral cancer

Smoking units/day	Predicted RR	Lower CI	Upper CI	RR Change per unit
0	0.97	0.53	1.60	NA
5	1.46	1.01	2.10	0.49
10	2.19	1.75	2.74	0.73
20	4.54	2.74	7.54	2.35
30	5.98	3.37	10.60	1.44
40	4.98	3.49	7.13	-1
50	3.84	2.41	6.13	-1.14

### Duration of smoking and oral cancer risk

**Fig. 3:**
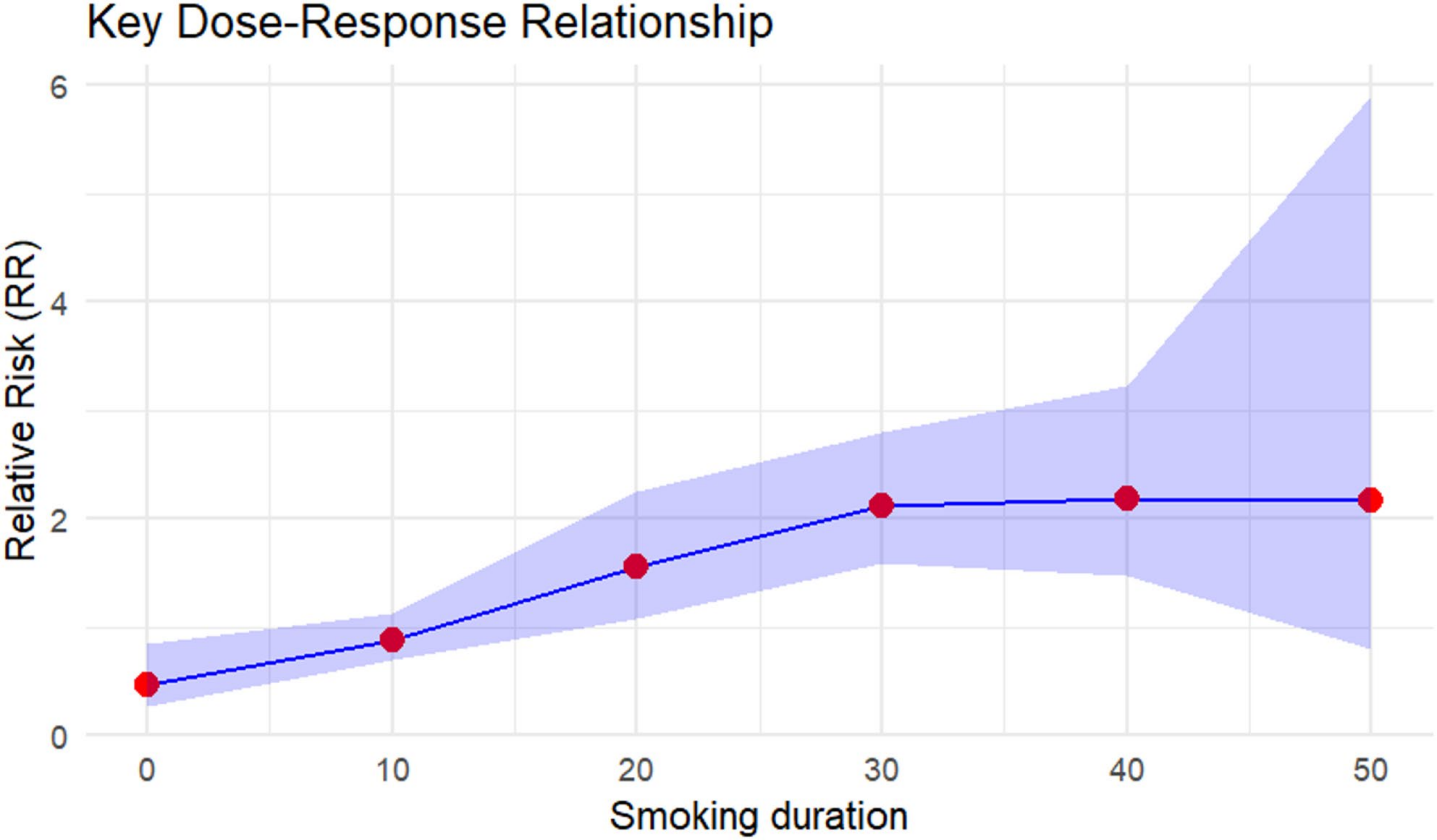
Dose-response relationship between smoking duration and oral cancer risk. Shaded areas indicate 95% confidence intervals

Twenty-two studies were included in the analysis of smoking duration. A statistically significant positive association was observed (QM [df = 2] = 49.75, *p* < 0.0001). However, comparison with the linear model showed no meaningful improvement in fit (AIC 188.6 vs. 189.1), and the additional spline term was not statistically significant (*p* = 0.249), indicating no evidence of departure from linearity. Risk increased progressively with longer duration, reaching an RR of 2.19 (95% CI: 1.48–3.23) at 40 years of smoking. The apparent slight decline at 50 years (RR = 2.16; 95% CI: 0.79–5.90) was accompanied by wide confidence intervals, reflecting limited precision at higher exposure levels (Fig. 3; Table 3). Between-study heterogeneity was substantial (QE [df = 75] = 339.68, *p* < 0.0001, I^2^ = 78%), but publication bias was not statistically significant (Kendall’s tau = 0.144, *p* = 0.062).

**Table 3:** Predicted RR for the duration of smoking and the risk of oral cancer

Duration in years	Predicted RR	Lower CI	Upper CI	RR change per unit
0	0.47	0.26	0.84	NA
5	0.64	0.44	0.94	0.17
10	0.88	0.69	1.12	0.24
20	1.56	1.08	2.25	0.68
30	2.11	1.60	2.79	0.55
40	2.19	1.48	3.23	0.08
50	2.16	0.79	5.90	-0.03

### Duration of alcohol consumption and oral cancer risk

**Fig. 4:**
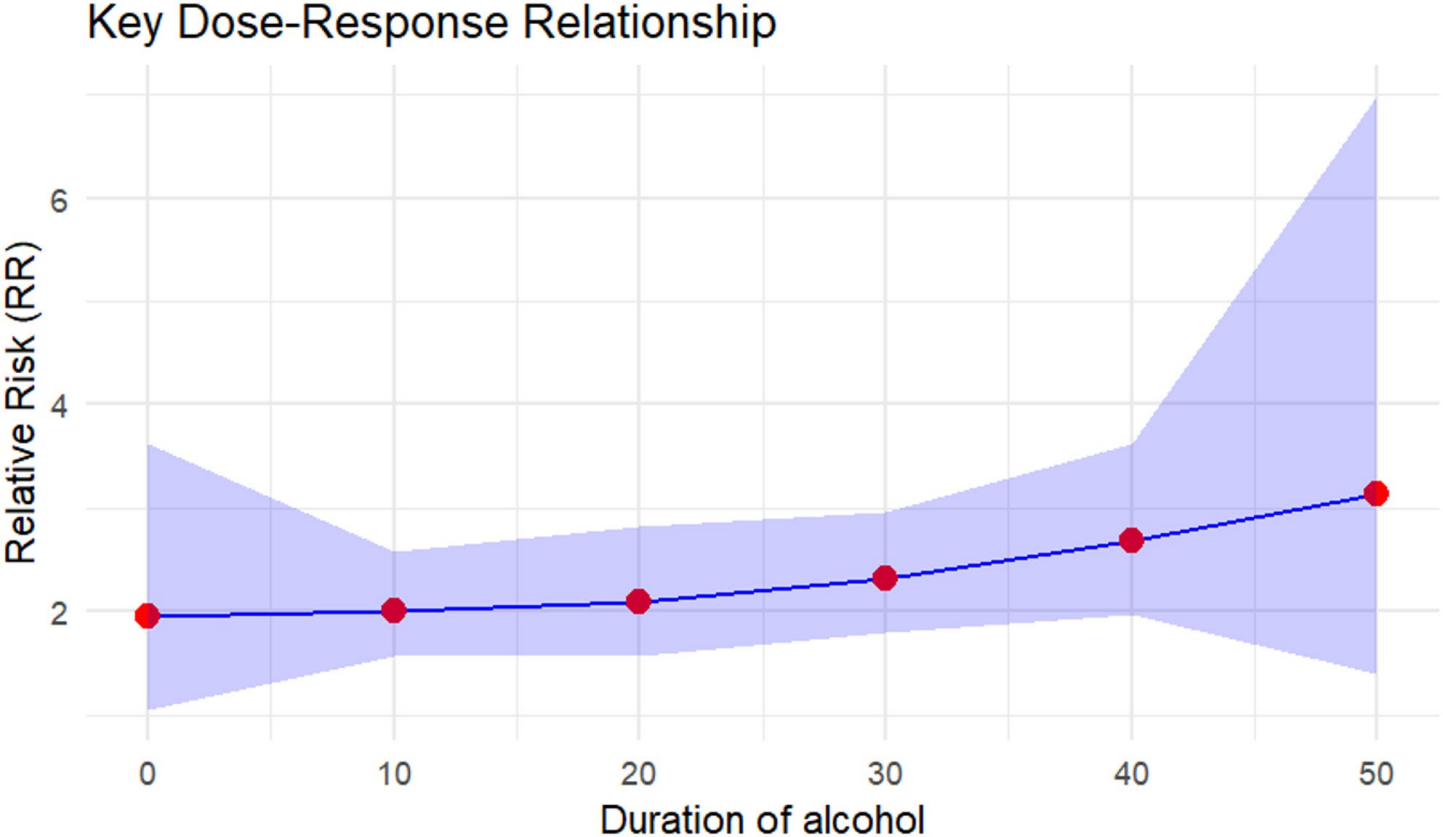
Dose-response relationship between duration of alcohol consumption and oral cancer risk. Shaded areas indicate 95% confidence intervals

Eleven studies assessed the association between alcohol use duration and oral cancer risk. No evidence of a non-linear association was observed (QM [df = 2] = 3.41, *p* = 0.182). The linear model demonstrated a better fit based on AIC and indicated a positive, though borderline, association (QM [df = 1] = 3.54, *p* = 0.060). The highest predicted risk was observed at 50 years of alcohol consumption (RR = 3.13; 95% CI: 1.40–6.97) (Fig. 4; Table 4). Moderate residual heterogeneity was present (QE [df = 29] = 54.98, *p* = 0.0025, I² = 47.3%), and no significant publication bias was detected (Kendall’s tau = − 0.024, *p* = 0.860).

**Table 4:** Predicted RR for the duration of alcohol drinking and the risk of oral cancer

Duration in years	Predicted RR	Lower CI	Upper CI	RR change per unit
0	1.94	1.04	3.62	NA
10	2.01	1.56	2.58	0.07
20	2.10	1.56	2.81	0.09
30	2.31	1.80	2.96	0.21
40	2.67	1.97	3.63	0.36
50	3.13	1.40	6.97	0.46

### Age of smoking initiation and oral cancer risk

**Fig. 5:**
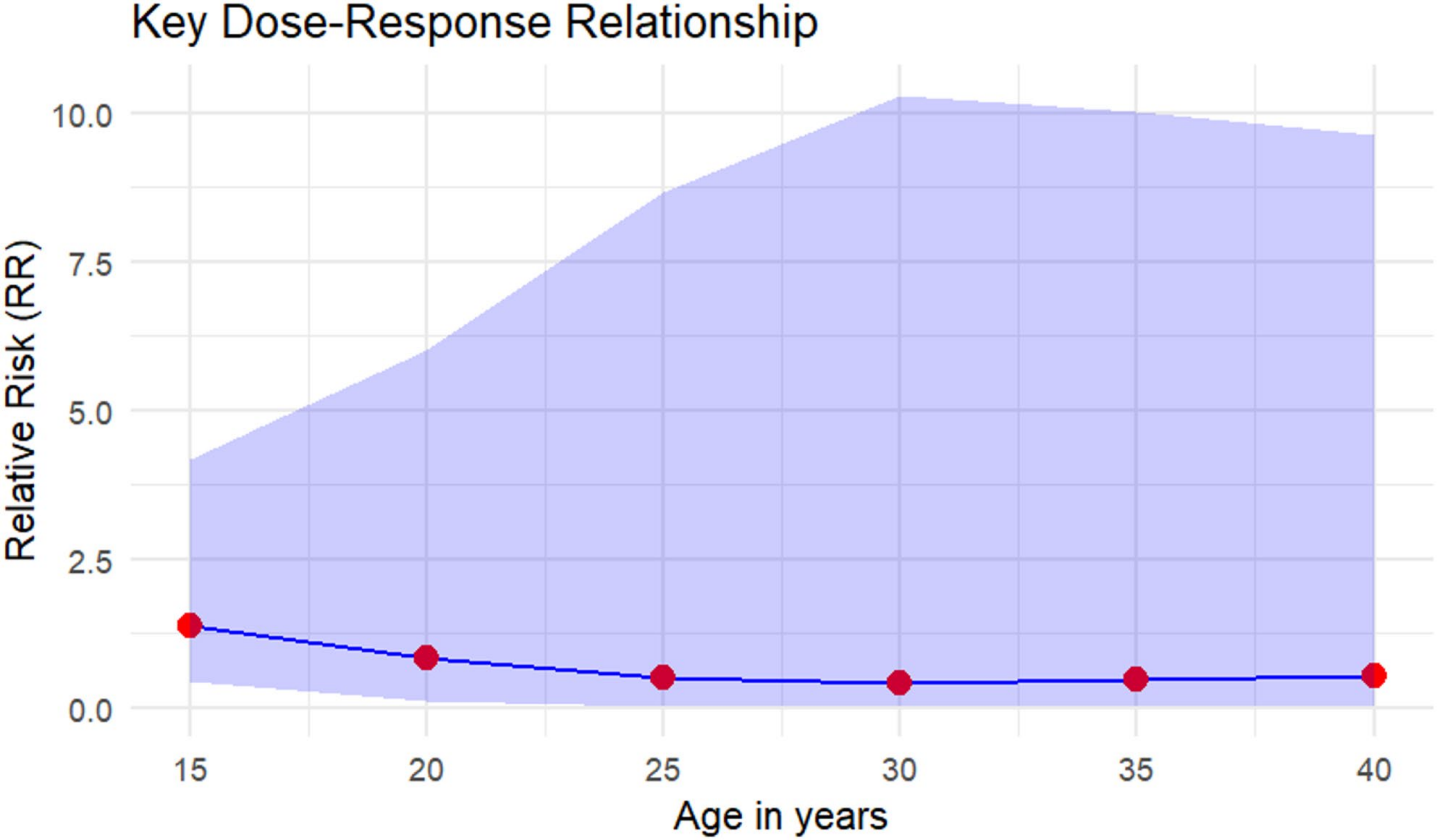
Dose-response relationship between age at smoking initiation and oral cancer risk. Shaded areas indicate 95% confidence intervals

The association between the age of smoking initiation and oral cancer risk was not statistically significant. The dose-response curve showed a declining trend in RR with increasing age of initiation, but the trend did not reach statistical significance. Neither a linear (QM [df = 1] = 0.00, *p* = 0.996) nor a non-linear association (QM [df = 2] = 1.20, *p* = 0.548) was observed. Substantial residual heterogeneity was present (QE [df = 22] = 123.34, *p* < 0.0001, I² = 82.2%). The predicted RR at age 15 was 1.37 (95% CI: 0.45–4.16), which declined to 0.42 (95% CI: 0.01–10.28) by age 30, and slightly increased thereafter (Fig. 5 and Table 5). Due to the wide confidence intervals and non-significant moderator effects, age of initiation does not appear to be an independent predictor of oral cancer risk. However, it may interact with other smoking variables, such as frequency and duration of smoking, to influence risk. No significant publication bias was detected (Kendall’s tau = -0.073, *p* = 0.6273).

**Table 5:** Predicted RR for the age of initiation of smoking and the risk of oral cancer

Age of smoking initiation	Predicted RR	Lower CI	Upper CI	RR change per unit
15	1.37	0.45	4.16	NA
20	0.82	0.11	6.01	-0.55
25	0.51	0.03	8.68	-0.31
30	0.42	0.01	10.28	-0.09
35	0.46	0.02	10.02	0.04
40	0.53	0.02	9.63	0.07

## Discussion

This dose-response meta-analysis demonstrates a significant association between tobacco smoking and oral cancer risk, with differing exposure-response patterns across smoking metrics. Smoking frequency exhibited a statistically significant departure from linearity, whereas smoking duration was adequately described by a linear trend. For duration of alcohol consumption, a weak positive linear association was suggested. In contrast, age at smoking initiation was not independently associated with oral cancer risk in dose-response analyses. These findings indicate that the shape and strength of the exposure-risk relationship vary by exposure metrics. The risk of oral cancer increased with increasing smoking units per day and longer smoking duration. However, an unexpected decline in the predicted RR was observed beyond an intensity of 30 cigarettes per day. This apparent decline could be attributed to a few methodological issues and the natural course of exposure and diseases. There was a paucity of epidemiological studies that provide precise risk estimates at very high levels of tobacco usage. Most of the included studies broadly categorised smoking exposure (e.g., 20 + cigarettes/day) and rarely reported granular estimates beyond 30 cigarettes/day. Consequently, the model’s predictions at higher levels of smoking relied on limited data, leading to wider confidence intervals and less stable risk estimates. This limited representation undermines the accuracy of risk extrapolation and may contribute to an artificial risk plateau or decline. A small number of studies included in this meta-analysis also reported a decrease in RR after 30 cigarettes/day [38, 54, 56, 58, 59]. However, these observed declines were not explained within the individual studies themselves. These findings may reflect methodological limitations, such as survivor bias, or underreporting of cigarette consumption among heavy smokers, or the development of other disorders due to chronic smoking. We believe, these unexplained patterns in individual studies contributed to the overall shape of the pooled dose-response curve.

A similar dose-response analysis of smoking and oropharyngeal cancer, published recently, revealed a non-linear increase in risk with smoking intensity, rising sharply between 6 [1.98 (95% CI: 1.74–2.25)], 10 [2.88 (95% CI: 2.37–3.50)], and 20 [4.78 (95% CI: 3.64–6.27)] cigarettes per day before plateauing [60]. A non-linear trend was also observed for smoking duration, with RRs of 2.01 (95% CI: 1.62–2.51) after seven years, 2.64 (95% CI: 1.96–3.57) after ten years, and 5.10 (95% CI: 3.27–7.96) after 20 years of exposure [60]. Consistent with these findings, our analyses also showed plateauing effects and wider confidence intervals at higher exposure levels for smoking duration and alcohol consumption, likely reflecting limited data availability at extreme exposure ranges and highlighting the need for more detailed exposure quantification in future studies.

The correlation between the age of initiation of smoking and the risk of oral cancer was found to be statistically nonsignificant, implying that it cannot be an independent factor in determining the risk of oral cancer. When combined with increased intensity and duration, the age of initiation of smoking could contribute to a higher risk.

**Fig. 6:**
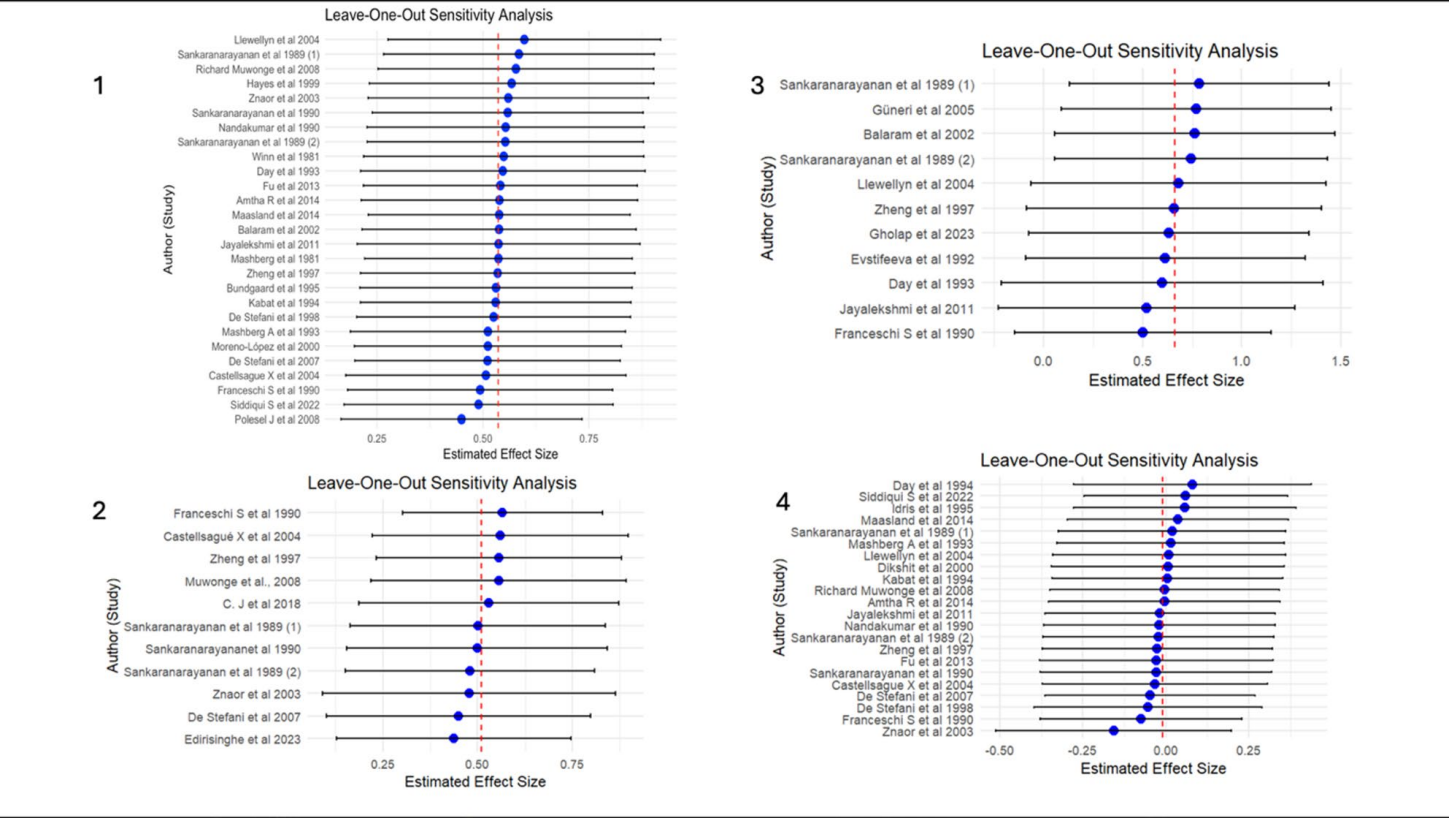
Sensitivity analysis: 1. Frequency of smoking tobacco and risk of oral cancer; 2. Duration of drinking alcohol and risk of oral cancer; 3. Age of initiation of smoking tobacco and risk of oral cancer; 4. Duration of smoking tobacco and risk of oral cancer. The results of all four sensitivity analyses, examining different exposure variables, indicate that no individual study had a significant impact on the overall pooled estimate, suggesting that the findings are robust

Subgroup analyses based demographic variables were not possible due to limitations in the primary studies. Although a relatively large number of studies were included, with some having substantial sample sizes, considerable heterogeneity was detected. As with most meta-analyses of observational studies, the pooled estimates reflect study-specific adjusted models, and the covariates included varied across studies. Differences in confounder adjustment, particularly regarding mutual control for smoking and alcohol exposures, may have contributed to residual heterogeneity and should be considered when interpreting the findings. In a few studies, details of controlled variables were not reported (Refer Table 1). Reducing heterogeneity through more consistently reported and well-adjusted models will be an important direction for future dose-response meta-analyses. Sensitivity analyses demonstrated that the overall dose-response relationships were robust, with no single study exerting undue influence on the pooled estimates. The stability of the models across leave-one-out analyses supports the reliability of the observed associations despite substantial heterogeneity. A detailed presentation of the sensitivity analyses for each exposure variable is provided in Fig. 6.

The studies included have all demonstrated a positive correlation between the frequency and duration of exposure to smoking tobacco and alcohol and the likelihood of developing oral cancer. Previous meta-analytic evidence has shown a strong dose-risk relationship between alcohol consumption and oral and pharyngeal cancer, with pooled relative risks of 1.21 for ≤ 1 drink/day and 5.24 for ≥ 4 drinks/day, and dose-response estimates rising from RR 1.29 at 10 g ethanol/day to RR 13.02 at 125 g ethanol/day [61]. Our findings extend this body of evidence by providing quantitative estimates of risk across duration of alcohol exposure. Dose-response meta-analyses are particularly valuable in elucidating such relationships, as they enable the identification of critical exposure thresholds and nonlinear risk patterns. To our knowledge, this is among the first dose-response meta-analyses to model the risk of oral cancer specifically regarding both smoking tobacco and alcohol consumption. Previously, a similar dose-response analysis was performed to quantify the risk of nasopharyngeal cancer and cigarette smoking with risk increasing linearly with both smoking intensity and duration among current smokers. Relative risks were 1.47 (95% CI: 1.16–1.84) for 10 cigarettes/day and 2.14 (95% CI: 1.35–3.40) for 20 cigarettes/day, whereas risks rose to 1.44 (95% CI: 1.04–1.99) after 20 years and 2.06 (95% CI: 1.08–3.94) after 40 years of smoking [62].

Our findings reinforce the strong association between smoking, alcohol consumption, and oral cancer risk, while also highlighting the clear dose-response relationship between exposure and disease. Such dose-response evidence is a defining criterion used by the International Agency for Research on Cancer (IARC) in evaluating human carcinogens, providing critical insights into both the magnitude and trajectory of risk across varying levels of exposure. These results carry important implications at both clinical and public health levels. Clinically, they can help identify individuals at elevated risk and guide the design of tailored preventive interventions. From a public health standpoint, recognising exposure thresholds enables more precise awareness campaigns and screening strategies, particularly in high-incidence regions, ultimately contributing to a reduction in the burden of oral cancer.

### Limitations

A major limitation of this meta-analysis is the substantial heterogeneity among included studies in the definition and quantification of exposure variables, which may have led to a more generalised pooled estimate. Part of this heterogeneity arises from differences in exposure categorisation; for instance, some studies combined current and ever users, which may have resulted in underestimation of risk, as the “ever user” category can include former users. This underscores the importance of accurately reporting and clearly categorising study populations in primary studies. The observed heterogeneity is also partly attributable to inconsistent adjustments for confounders across studies, which limits comparability and precludes subgroup analyses. Additionally, sparse data at higher exposure levels reduced the precision of risk estimates in the upper range, and potential biases, including survivor bias, underreporting among heavy users, and variation in exposure assessment methods, cannot be excluded. The dose-response analyses were typically reported as consolidated estimates across smoking products rather than stratified by product type. As a result, a further limitation in our analysis is lack of product-specific dose-response meta-analyses (e.g., for cigarettes alone) which was not feasible. Standardised cumulative exposure metrics such as pack-years and alcohol consumption in grams per day were not analysed because primary studies reported heterogeneous and non-comparable exposure definitions, highlighting an important methodological gap that would enable more refined dose-response assessment. Future studies should standardise exposure measurement, ensure comprehensive confounder adjustment, and provide granular data across the full exposure spectrum to refine dose-response estimates. Another drawback of this analysis is that the interaction between the different exposure variables could not be accounted for in this review, given the complexity of studying the RRs with different combinations of exposure characteristics to two different risk factors, and the sparsity of primary studies that would have accounted for such interaction at the time of primary analysis.

## Conclusions

This dose-response meta-analysis demonstrates strong non-linear and independent associations between the smoking frequency and smoking duration and the risk of oral cancer, as well as a positive, although weaker, association between the duration of alcohol consumption and oral cancer. These findings identify exposure-specific thresholds at which risk markedly increases, underscoring the importance of precise exposure assessment in clinical risk stratification and public health interventions. While estimates at the highest exposure levels should be interpreted cautiously because of sparse data, the observed trends highlight the need for prevention strategies tailored to the intensity and duration of use.

Future research should prioritise standardised exposure measurement, consistent adjustment for confounders, and more granular reporting of high-exposure categories to refine these estimates. Incorporating these data into screening guidelines could improve early detection and reduce the global burden of oral cancer, particularly in high-incidence regions.

## Supplementary Information

Supplementary Material 1.

Supplementary Material 2.

Supplementary Material 3.

## Data Availability

All data generated or analysed during this study are provided in the tables, figures and supplementary materials of this published article.
