# Supplementary material for: Evaluation of dose-response relationships between smoking tobacco, alcohol consumption and oral cancer: a systematic review and meta-analysis
Source: BMC Public Health. 2026 May 22;26:2148. doi: 10.1186/s12889-026-27796-1 (PMC13371301; doi:10.1186/s12889-026-27796-1)
Supplement: Supplementary file 1 — Supplementary Material 1. [file 12889_2026_27796_MOESM1_ESM.docx]

**SEARCH STRATEGY**

The search strategy for the systematic review and meta-analysis was structured around three key concepts, which were systematically combined using appropriate Boolean operators. These included:

1. **Oral Cancer** - terms covering “oral squamous cell carcinoma,” “oral cavity neoplasms,” “mouth neoplasms,” and related synonyms.
2. **Risk Factors** - exposure related terms for smoking tobacco (cigarettes, bidis, cigars, pipes), and alcohol related terms.
3. **Study Design** - epidemiological study types such as “case-control,” “cohort,” and “cross-sectional.”

These three concepts, along with the synonyms for risk estimates like “relative risk”, “odds ratio”, etc., were applied uniformly across all selected electronic databases (PubMed, EMBASE, Scopus, Web of Science, Cochrane Central, and ProQuest), ensuring consistency and reproducibility in identifying relevant articles. Both controlled vocabulary (e.g., MeSH terms in PubMed) and free-text keywords were incorporated to maximize retrieval sensitivity and specificity.

PUBMED: 3501 articles

|  | #1 AND #2 AND #3 AND #4 |
| --- | --- |
| 1 | (((oral cancer OR "oral squamous cell carcinoma" OR "oral malignan*" OR "oral neoplas*" OR "mouth cancer" OR "mouth neoplas*" OR mouth malignan* OR "oral cavity cancer" OR "oral cavity neoplas*" OR "oral cavity malignan*" OR "tongue cancer" OR "tongue neoplas*" OR "tongue malignan*" OR palat* neoplas* OR palat* cancer OR palat* malignan* OR gingiv* neoplas* OR gingiv* cancer OR gingiv* malignan* OR "gum cancer" OR gum neoplas* OR gum malignan* OR buccal malignan* OR buccal neoplas* OR "buccal cancer" OR "cheek cancer" OR cheek neoplas* OR cheek malignan* OR "lip neoplas*" OR "lip cancer" OR "lip malignan*" OR "labial neoplas*" OR "labial cancer" OR labial malignan* OR "Mouth Neoplasms"[Mesh] OR oral potentially malignan* disease* OR "oral potentially malignant disorder*" OR "oral premalignant condition*" OR "premalignant condition*" OR "precancerous condition*" OR "oral precancerous condition*" OR "precancerous lesion*" OR potentially malignan* lesion* OR premalignan* lesion* OR leukoplakia OR erythroplakia OR "oral submucous fibrosis" OR OSF OR OSMF OR OPMD* OR "oral lichen planus" OR erythroleukoplakia OR "oral lichenoid reaction" OR "oral lichenoid lesion*" OR "Leukoplakia, Oral"[Mesh] OR "Oral Submucous Fibrosis"[Mesh] OR "Lichen Planus, Oral"[Mesh]) |
| 2 | (((Smoking[Mesh] OR (cig[Text Word]) OR (cigarette [Text Word]) OR (smok*[Text Word]) OR (bidi[Text Word]) OR (alcohols[MeSH Terms]) OR (alcohol drinking[MeSH Terms]) OR (ethanol[Text Word]) OR (alcohol[Text Word])) OR (tobacco smoking[MeSH Terms]))) |
| 3 | (((case control studies[MeSH Terms]) OR (cohort studies[MeSH Terms]) OR (cross sectional studies[MeSH Terms]) OR (epidemiological studies[MeSH Terms]) OR ("longitudinal stud*"[Text Word]) OR ("case-control"[Text Word]))))) |
| 4 | ("risk ratio" OR "relative risk" OR risk OR "odds ratio" OR "risk estimate" OR (risk factor[MeSH Terms]) OR (risk factors[MeSH Terms]) |
