# Supplementary material for: Evaluation of dose-response relationships between smoking tobacco, alcohol consumption and oral cancer: a systematic review and meta-analysis
Source: BMC Public Health. 2026 May 22;26:2148. doi: 10.1186/s12889-026-27796-1 (PMC13371301; doi:10.1186/s12889-026-27796-1)
Supplement: Supplementary file 2 — Supplementary Material 2. [file 12889_2026_27796_MOESM2_ESM.docx]

**RISK OF BIAS: NEWCASTLE-OTTAWA QUALITY ASSESSMENT SCALE**

1. **CASE-CONTROL STUDIES:**

| **Author** | **Selection** | | | | **Comparability** | **Exposure** | | | **Total** |
| --- | --- | --- | --- | --- | --- | --- | --- | --- | --- |
|  | **Case Definition** | **Representativeness of case** | **Selection of Controls** | **Definition of Controls** |  | **Ascertainment of exposure** | **Same method of ascertainment for cases and controls** | **Non-response rate** |  |
| Day et al., 1993 (31) | 1 | 1 | 1 | 1 | 2 | 0 | 1 | 0 | 7 |
| Mashberg et al., 1993 (30) | 1 | 1 | 1 | 1 | 2 | 0 | 1 | 0 | 7 |
| Hayes et al., 1999 (36) | 1 | 1 | 1 | 1 | 2 | 0 | 1 | 0 | 7 |
| Dikshit et al., 2000(50) | 1 | 1 | 1 | 1 | 2 | 0 | 1 | 0 | 7 |
| Castellsague et al., 2004 (38) | 1 | 1 | 0 | 1 | 2 | 0 | 1 | 1 | 7 |
| De Stefani et al., 2007 (53) | 1 | 1 | 0 | 1 | 2 | 0 | 1 | 1 | 7 |
| Polosel et al., 2008 (40) | 1 | 1 | 1 | 1 | 1 | 0 | 1 | 1 | 7 |
| Muwonge et al., 2008 (12) | 1 | 1 | 1 | 1 | 2 | 0 | 1 | 0 | 7 |
| Fu et al., 2013 (41) | 1 | 1 | 0 | 1 | 2 | 0 | 1 | 1 | 7 |
| Mashberg et al., 1981 (26) | 1 | 1 | 0 | 1 | 2 | 0 | 1 | 0 | 6 |
| Sankarnarayanan et al., 1989 [1](46) | 1 | 1 | 0 | 1 | 2 | 0 | 1 | 0 | 6 |
| Sankarnarayanan et al., 1989 [2] (27) | 1 | 1 | 0 | 1 | 2 | 0 | 1 | 0 | 6 |
| Franceschi et al., 1990 (28) | 1 | 1 | 0 | 1 | 2 | 0 | 1 | 0 | 6 |
| Nandakumar et al., 1990 (29) | 1 | 1 | 0 | 1 | 2 | 0 | 1 | 0 | 6 |
| Sankarnarayanan et al., 1990 (47) | 1 | 1 | 0 | 1 | 2 | 0 | 1 | 0 | 6 |
| Kabat et al., 1994 (32) | 1 | 1 | 0 | 1 | 2 | 0 | 1 | 0 | 6 |
| Bundgard et al., 1995(33) | 1 | 1 | 0 | 1 | 2 | 0 | 1 | 0 | 6 |
| Idris et al., 1995 (48) | 1 | 1 | 1 | 1 | 1 | 0 | 1 | 0 | 6 |
| Zheng et al., 1997 (34) | 1 | 1 | 0 | 1 | 2 | 0 | 1 | 0 | 6 |
| De Stefani et al., 1998 (35) | 1 | 1 | 0 | 1 | 2 | 0 | 1 | 0 | 6 |
| Balaram et al., 2002 (37) | 1 | 1 | 0 | 1 | 2 | 0 | 1 | 0 | 6 |
| Znaor et al., 2003 (51) | 1 | 1 | 0 | 1 | 2 | 0 | 1 | 0 | 6 |
| Llewellyn et al., 2004 (39) | 1 | 1 | 0 | 1 | 2 | 0 | 1 | 0 | 6 |
| Amtha et al., 2014 (42) | 1 | 1 | 0 | 1 | 2 | 0 | 1 | 0 | 6 |
| Gholap et al., 2023 (45) | 1 | 1 | 0 | 1 | 2 | 0 | 1 | 0 | 6 |
| Edirisinghe et al., 2023 (44) | 1 | 1 | 1 | 1 | 1 | 0 | 1 | 0 | 6 |
| Winn et al., 1981 (25) | 1 | 1 | 0 | 1 | 1 | 0 | 1 | 0 | 5 |
| C. J et al., 2018 (43) | 1 | 1 | 0 | 1 | 1 | 0 | 1 | 0 | 5 |
| Morenzo-Lopez et al., 2000(49) | 1 | 1 | 0 | 1 | 0 | 0 | 1 | 0 | 4 |
| Guneri et al., 2005 (52) | 1 | 1 | 0 | 1 | 0 | 0 | 1 | 0 | 4 |
| Siddiqui et al., 2024 (54) | 1 | 1 | 0 | 1 | 0 | 0 | 1 | 0 | 4 |

1. **COHORT STUDIES:**

| **Author** | **Selection** | | | | | **Comparability** | | **Outcomes** | | | **Total** |
| --- | --- | --- | --- | --- | --- | --- | --- | --- | --- | --- | --- |
|  | **Representativeness of exposed cohort** | **Selection of non-exposed cohort** | **Ascertain--ment of exposure** | **Outcome not present at the start of the study** |  | | **Assessment of outcomes** | | **Length of follow-up satisfactory** | **Adequacy of follow-up** |  |
| Jayalekshmi et al., 2011(55) | 1 | 1 | 1 | 1 | 2 | | 1 | | 1 | 1 | 9 |
| Maasland et al., 2014(56) | 1 | 1 | 1 | 1 | 2 | | 1 | | 1 | 1 | 9 |

1. **CROSS-SECTIONAL STUDY:**

| **Author** | **Selection** | | | | **Comparability** | **Outcome** | |  |
| --- | --- | --- | --- | --- | --- | --- | --- | --- |
|  | **Representativeness of the sample** | **Sample size** | **Non-respondents** | **Ascertainment of the exposure (risk factor)** |  | **Assessment of outcome** | **Statistical test** | **Total** |
| Evstifeeva et al., 1992 (57) | 1 | 1 | 0 | 1 | 2 | 1 | 1 | 7 |
