# Supplementary material for: Evaluation of dose-response relationships between smoking tobacco, alcohol consumption and oral cancer: a systematic review and meta-analysis
Source: BMC Public Health. 2026 May 22;26:2148. doi: 10.1186/s12889-026-27796-1 (PMC13371301; doi:10.1186/s12889-026-27796-1)
Supplement: Supplementary file 3 — Supplementary Material 3. [file 12889_2026_27796_MOESM3_ESM.docx]

**HETROGENEITY AND PUBLICATION BIAS**:

| **Factor** | **Exposure Variables Included (k)** | **Kendall's Tau (Publication Bias)** | |
| --- | --- | --- | --- |
| **Smoking Frequency** | 86 | 0.087 (p = 0.236) | 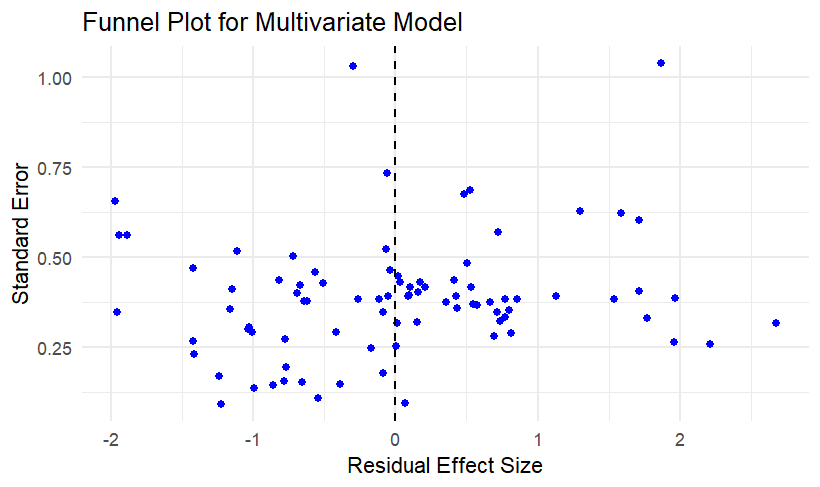  Fig 1: Funnel Plot for Smoking Frequency |
| **Smoking Duration** | 78 | 0.144 (p = 0.062) | 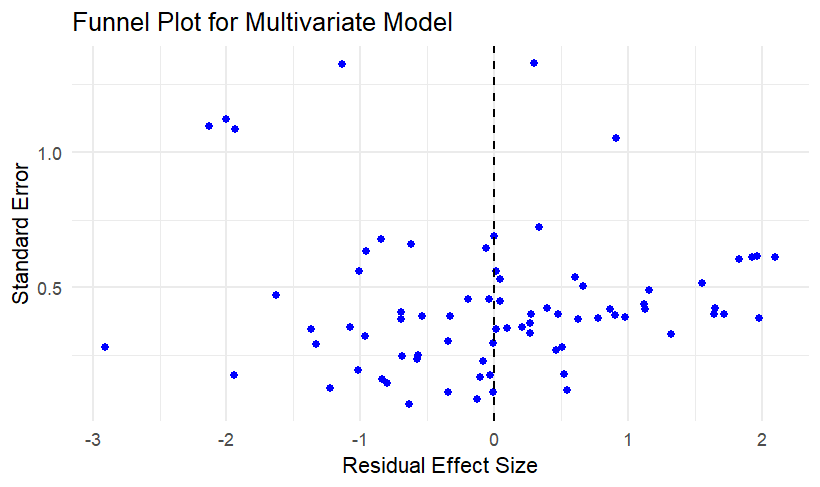  Fig 2: Funnel Plot for Smoking Duration |
| **Alcohol Consumption Duration** | 32 | -0.024 (p = 0.860) | 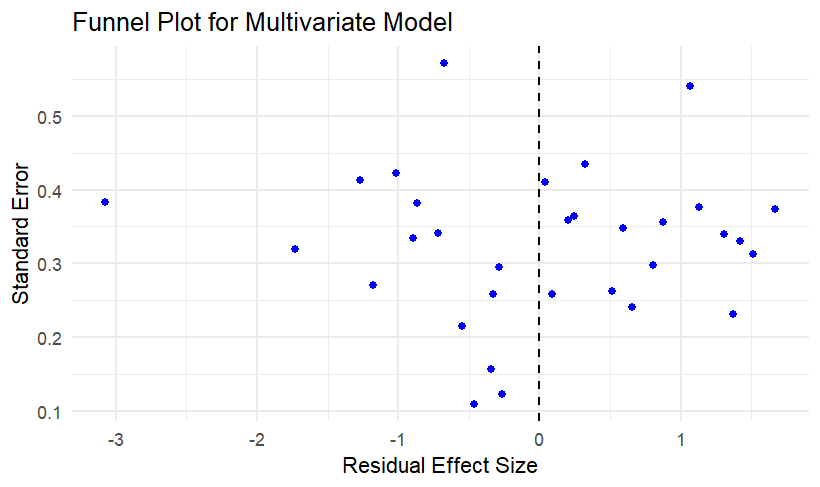  Fig 3: Funnel Plot for Duration of Alcohol Consumption |
| **Age of Smoking Initiation** | 25 | -0.073 (p = 0.6273) | **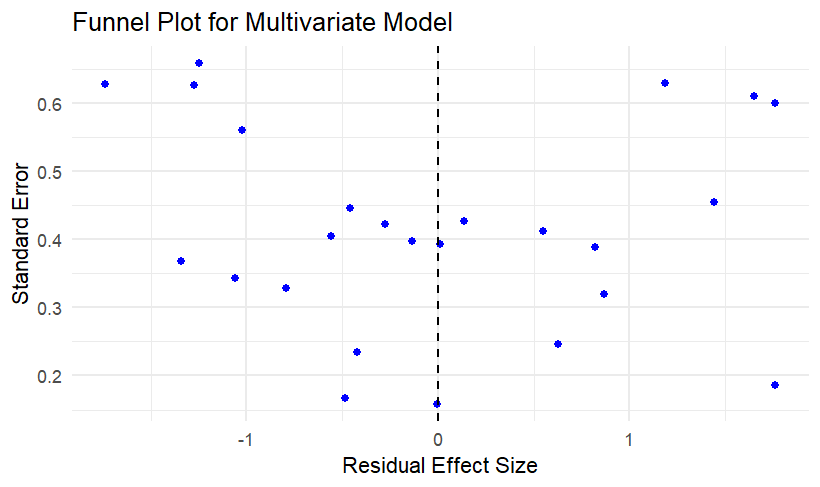**  Fig 4: Funnel Plot for Age of smoking Initiation |

*k* represents the number of exposure variables included in the dose-response meta-analysis. Publication bias was evaluated using Kendall’s Tau rank correlation; *p-values* < 0.05 suggest potential bias.
